# Qualitative and quantitative evaluation of acute angle-closure mechanisms

**DOI:** 10.1186/s12886-017-0635-8

**Published:** 2017-12-11

**Authors:** Yanin Suwan, Sunpong Jiamsawad, Apichat Tantraworasin, Lawrence Geyman, Wasu Supakontanasan, Chaiwat Teekhasaenee

**Affiliations:** 1 From the Department of Ophthalmology, Faculty of Medicine, Ramathibodi Hospital, Mahidol University, Bangkok, Thailand; 2 From General Thoracic Surgery Unit, Department of Surgery, Faculty of Medicine, Chiang Mai University, Chiang Mai, Thailand; 3 Icahn School of Medicine at Mount Sinai, New York, NY USA

**Keywords:** Acute angle-closure, Angle-closure, Ultrasound biomicroscopy, Biometry

## Abstract

**Background:**

To evaluate ocular biometric parameters in different subtypes of acute angle closure and compared to fellow eyes of AAC and PACS eyes.

**Methods:**

This is a retrospective chart review study. A total of 167 eyes (96 patients) consisting of 71 AAC eyes, 71 fellow eyes of AAC, and 25 PACS eyes were recruited. All patients underwent ocular examination and biometry. The mechanism of AAC was confirmed by ultrasound biomicroscopy. We then subdivided AAC eyes into four subgroups: crowded-angle (CR), lens subluxation (LS) pupillary block (PB), and plateau iris syndrome (PL). Outcome variables included anterior chamber depth (ACD), lens thickness (LT), vitreal length (VL), axial length (AL), lens position and relative lens position (LP and RLP, respectively), and lens axial length factor (LAF).

**Results:**

Among the three groups, ACD was shallower in AAC eyes than fellow eyes of AAC and PACS eyes (*p* < 0.01 for both) and AAC eyes demonstrated a lesser LP and RLP. The LT, VL, AL, and LAF were not significantly different among the three groups. Among the four subgroups, LS displayed the most shallow ACD (*p* = 0.01). The lens position in PL was greater than in CR and LS (*p* < 0.05 and <0.01, respectively).

**Conclusions:**

AAC eyes had a more anterior lens position than fellow eyes and PACS eyes, though lens thickness did not differ among the groups. As such, an anterior lens position may offer more sensitive prognostication regarding future development of AAC compared to lens thickness.

## Background

Acute angle-closure (AAC) is a potentially blinding ophthalmic emergency that requires prompt treatment to markedly lower the increased intraocular pressure (IOP) [1]. Acute primary angle-closure is a bilateral, asymmetrical condition that frequently involves the fellow eye [2]. Several ocular biometric parameters have been identified for angle-closure development, such as small corneal diameter [2], small radius of the anterior and posterior corneal curvature [2], short axial length, shallow central and peripheral anterior chamber depth [3, 4], thick and anteriorly positioned lens [5–8], and a large lens axial length factor [9, 10].

Ultrasound biomicroscopy (UBM) can be used to determine the mechanisms of AAC by demonstrating the relationship between the peripheral iris, ciliary body, and trabecular meshwork [11]. UBM can also demonstrate both static and dynamic images of the anterior chamber. In contrast, although anterior segment optical coherence tomography visualizes the angle well, it generally cannot sufficiently image the ciliary body. This limitation precludes appreciation of a plateau iris (particularly if the cornea is edematous, thereby precluding gonioscopy).

There have been several studies comparing biometric parameters among AAC, fellow eyes, or primary angle-closure suspect (PACS) eyes in an attempt to identify risk factors that may contribute to an acute episode [5, 7, 12–15]. However, to our knowledge, few studies have analyzed the differences in biometric parameters between distinct mechanisms of acute angle-closure. We recently reported mechanisms of AAC classified by UBM.

A study of the biometric parameters associated with angle-closure will further expand our understanding regarding pathologic mechanisms and guide us toward more effective diagnoses and treatments.

## Methods

This study was conducted by retrospective chart review of consecutive patients who presented with the diagnosis of either AAC or PACS to the Department of Ophthalmology, Ramathibodi Hospital, Mahidol University, Thailand, from June 2011 to February 2015. This study was approved by Ramathibodi Hospital, Mahidol University Ethic Committee. The study was Health Insurance Portability and Accountability Act compliant and adhered to the tenets of the Declaration of Helsinki. Written informed consent was obtained from all subjects. We utilized UBM to determine the mechanism of angle-closure in 71 AAC eyes, 71 fellow eyes of AAC, and 25 PACS eyes. Each patient also was examined by A-scan ultrasound biometry.

Three groups were examined.

1. AAC was defined as: (1) presence of at least one of the following symptoms: periocular pain or headache, nausea and/or vomiting, decreased vision, and/or antecedent history of intermittent rainbow-colored halos around lights; (2) documentation of presenting IOP ≥ 21 mmHg (as measured by Goldmann applanation tonometry); (3) presence of iridotrabecular contact more than 180° on gonioscopy; and (4) slit-lamp biomicroscopic findings of at least four of the followings: ciliary injection, corneal epithelial edema, fixed mid-dilated pupil, glaukomflecken, and shallow peripheral anterior chamber.
2. A fellow eye of AAC was defined as: (1) presence of iridotrabecular contact of more than 180° on gonioscopy, and (2) no history or signs of previous AAC attack.
3. PACS was defined as: (1) presence of iridotrabecular contact more than 180° without PAS on gonioscopy; (2) the absence of a glaucomatous optic nerve and visual field damage; (3) no history or signs of a previous AAC attack; and (4) an IOP of <21 mmHg without medication.

Exclusion criteria included: (1) an open-angle (>180°) on gonioscopic examination before use of anti-glaucoma medication; (2) a history of laser peripheral iridoplasty laser pupilloplasty surgical peripheral iridectomy cataract or filtering surgery; (3) secondary angle-closure such as ocular trauma neovascularization of the iris uveitis or lens intumescence with significantly different cataract severity between eyes (i.e. phacomorphic glaucoma); (4) patients with cataract surgery or goniosynechialysis prior to UBM; and (5) cases of bilateral acute angle-closure.

Standard demographic and ophthalmic data were also recorded: age, sex, laterality of affected eye, presenting IOP, A-scan biometry (axial length (AL), anterior chamber depth (ACD), lens thickness (LT), vitreous length (VL), and UBM to evaluate the underlying mechanisms of AAC.

**Fig. 1:**
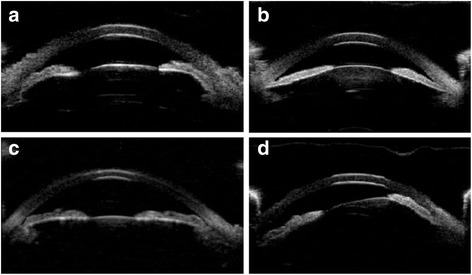
Ultrasound biomicroscopic images demonstrate angle-closure mechanisms in AAC. **a** pupillary block; **b** crowded-angle; **c** plateau iris, and **d** anterior lens subluxation

We classified all AAC subgroups into four categories: pupillary block (PB), crowded-angle (CR), anterior lens subluxation (LS), and plateau iris syndrome (PL) (Fig. 1).

PB was defined as: (1) characteristic iris bombé on gonioscopy, and (2) convex iris configuration on UBM with decreased iridolenticular contact under dark illumination in at least three quadrants.

PL was defined as: (1) iridotrabecular apposition in the presence of a patent iridectomy as revealed by gonioscopy, anteriorly and centrally angled peripheral iris, a flat or slightly convex iris, and/or the sign of a double hump signifying the formation of anterior concavity of the iris at the lens equator level and ciliary body on the indentation gonioscopy; (2) absence of pupillary block; (3) UBM diagnosis of PL with anteriorly directed ciliary body, an absent ciliary sulcus, a steep iris approach from its point of insertion followed by a downward angulation from the corneoscleral wall, presence of a central flat iris plane, iridotrabecular contact in at least three quadrants; and (4) presence of a patent iridotomy.

CR was defined as: (1) iridotrabecular contact more than 180° in the presence of visible ciliary processes and volcanic iris configuration on gonioscopic examination, and (2) UBM diagnosis indicating shallow central and peripheral anterior chamber depth and increased iridolenticular contact distance.

LS was defined as: (1) iridotrabecular contact more than 180° on gonioscopic examination and slit-lamp biomicroscopy showing phacodonesis, tilting of lens, and/or visible vitreous in anterior or posterior chamber; and (2) UBM diagnosis showing shallow central and peripheral anterior chamber depth, tilting of lens, asymmetrical ACD in the same eye, and/or asymmetrical iris configuration.

### A-scan ocular biometry

In this study, A-scan ultrasound biometry (OcuScan**®**RxP Ophthalmic Ultrasound, Alcon, Ft Worth Tx, USA) was used to measure AL, ACD, LT, and VL. Care was taken not to exert pressure on the cornea by using the immersion technique. Measurements were made by trained technicians and repeated until three successive values within 0.1 mm for ACD and 0.3 mm for AL were obtained. These data were used to calculate lens position (LP = ACD + $\frac{1}{2}$ LT), relative lens position (RLP = $\frac{\mathrm{LP}}{\mathrm{AL}}$ × 10), and lens axial length factor (LAF = $\frac{\mathrm{LT}}{\mathrm{AL}}$ × 10).

### Qualitative UBM analysis

The UBM measurements were performed by one of three experienced-glaucoma specialists (YS, CT, or WS) who used the UBM with a 50-MHz transducer probe (Aviso, Quantel Medical, Bozeman, MT, USA). The measurements were obtained at the 3, 6, 9, and 12 o’clock positions of both eyes of each subject in the supine position. UBM assessments were carried out in dark (2 lx) and bright (400 lx) illumination to determine the dynamic changes of the iridocorneal relationship. The most pronounced feature that contributed to AAC development, as determined by the agreement of two of the three glaucoma specialists was classified as the primary mechanism.

### Statistical analysis

Statistical analysis was performed using STATA software version 12 (StataCorp, College Station, Texas, USA). Continuous variables were analyzed using the analysis of variance (ANOVA) or Kruskal-Wallis test depending on data distribution. Categorical variables were analyzed by the Fisher Exact Probability test. Logistic regression analysis including checks for multicollinearity was performed. The dependent variable was AAC vs. PACS. Our sample size had a 100% power to detect 0.32-mm difference in ACD between AAC and PACS eyes using a type I error of 0.05. A *p*-value of less than 0.05 was considered statistically significant.

## Results

A total of 167 eyes (96 patients) with angle-closure were recruited, consisting of 71 AAC eyes, 71 fellow eyes of AAC, and 25 PACS eyes. Four fellow eyes were pseudophakic and were excluded from analysis. There was no significant difference in mean age between AAC/fellow eye subjects and PACS subjects (63.3 ± 7.5 vs. 62.5 ± 6.1 years, *p* = 0.90). Although there was a female preponderance in each group, there was no difference in gender distribution between the groups (*p* = 0.94).

In AAC eyes, the ACD was significantly shallower compared to each of the other two groups (*p* < 0.001 for both). However, there was no significant difference in ACD between fellow eyes of AAC and PACS eyes (*p* = 0.99) (Table 1).

**Table 1:** Demographic characteristics of AAC mechanisms

	AAC eyes				PACS eyes (N = 25)	*p*- value
	CR (*N* = 30)	LS (*N* = 19)	PB (*N* = 16)	PL (*N* = 6)		
Age (years)^a^	63.0 ± 7.2 [48–85]	62.0 ± 7.4 [51–81]	66.4 ± 8.2 [56–88]	60.2 ± 6.9 [50–69]	62.5 ± 6.1 [50–72]	0.276
Gender (female)(%)	23(76.7)	11(57.9)	14 (87.5)	6 (100.0)	18 (72)	0.284

*CR* crowded-angle, *LS* lens subluxation, *PB* pupillary, *PL* plateau iris syndrome

^a^Age reported as mean ± standard deviation [range]

The LP and RLP of AAC eyes were significantly lesser compared to each of the other two groups (*p* < 0.01 and <0.01, respectively). However, no significant difference was found in the LP and RLP between fellow eyes of AAC and PACS eyes (*p* = 0.35 and 0.95, respectively).

The LT, VL, AL, and LAF were not significantly different among the three groups.

Further analysis with logistic regression analysis confirmed statistical significance of ACD, LP, and RLP. Furthermore, though not statistically significant, all ocular biometric parameters in fellow eyes of AAC had slightly smaller ocular dimensions than the PACS eyes (*p* = 0.29, 0.93, and 0.24 for LT, VL, and AL, respectively).

AAC eyes were then subdivided into the following four subgroups based on gonioscopic findings and UBM: crowded-angle (CR), lens subluxation (LS), pupillary block (PB), and plateau iris syndrome (PL). There was no significant difference in age among the four subgroups (Table 2). However, a significant difference in gender was present among the four subgroups. The range of male-to-female ratios extended from the PL group, in which there were no male patients, to the LS group, in which the ratio was 1:1.3.

**Table 2:** Ocular Biometric Parameters of AAC eyes, Fellow Eyes of AAC, and PACS eyes

Biometric Parameters	PACS eyes (*N* = 25)	Fellow eyes of AAC (*N* = 67)	AAC eyes (*N* = 71)	*p*-value^*^	*p*-value^†^
ACD (mm)	2.1 ± 0.2	2.1 ± 0.4	1.8 ± 0.3	< 0.001	< 0.001
LT (mm)	5.2 ± 0.3	5.0 ± 0.6	5.1 ± 0.3	0.30	
VL (mm)	15.1 ± 0.8	15.0 ± 0.9	15.2 ± 1.1	0.35	
AL (mm)	22.8 ± 0.9	22.5 ± 0.9	22.6 ± 1.0	0.27	
LP	4.7 ± 0.2	4.6 ± 0.3	4.3 ± 0.3	< 0.001	< 0.001
RLP	2.0 ± 0.1	2.0 ± 0.1	1.9 ± 0.2	< 0.001	< 0.001
LAF	2.3 ± 0.2	2.2 ± 0.3	2.3 ± 0.2	0.76	

*AAC* acute angle-closure, *PACS* primary angle-closure suspect, *ACD* anterior chamber depth, *LT* lens thickness, *VL* vitreal length, *AL* axial length, *LP* lens position, *RLP* relative lens position, *LAF* lens axial length factor

^*^ *p*-value represents analysis of all three groups compared to each other (1-way ANOVA)

^†^Dependent variable is PACS vs. AAC. Regression analysis is adjusted for age and gender

The differences in ocular biometric parameters among each AAC mechanism were analyzed (Tables 3 and 4). The ACD was greater in the PL group compared to the CR and LS groups, though there was no significant difference in ACD among CR, LS, and PB. Overall, there was a trend toward a deeper ACD, and from shallow to deep, the subgroups ranked: LS, CR, PB, and PL.

**Table 3:** Ocular Biometric Parameters Among Each Acute Angle-Closure Mechanism

Biometric Parameters	CR (*N* = 30)	LS (*N* = 19)	PB (*N* = 16)	PL (*N* = 6)	*p*-value^*^
ACD (mm)	1.7 ± 0.2	1.6 ± 0.3	1.9 ± 0.3	2.2 ± 0.2	<0.001
AL (mm)	22.3 ± 0.6	23.0 ± 1.4	22.6 ± 0.8	22.7 ± 0.5	0.07
LT (mm)	5.2 ± 0.3	5.0 ± 0.4	5.0 ± 0.3	5.0 ± 0.3	0.19
VL	14.9 ± 0.7	15.9 ± 1.6	15.1 ± 1.0	15.1 ± 0.4	< 0.05
LP	4.3 ± 0.3	4.1 ± 0.3	4.4 ± 0.3	4.7 ± 0.2	<0.001
RLP	1.9 ± 0.1	1.8 ± 0.2	1.9 ± 0.1	2.1 ± 0.1	<0.001
LAF	2.3 ± 0.1	2.2 ± 0.2	2.2 ± 0.2	2.2 ± 0.2	< 0.05

*ACD* anterior chamber depth, *LT* lens thickness, *VL* vitreal length, *AL* axial length, *LP* lens position, *RLP* relative lens position, *LAF* lens axial length factor, *CR* crowded-angle, *LS* lens subluxation, *PB* pupillary block, *PL* plateau iris syndrome

^*^ *p*-value represents analysis of all four groups compared to each other (1-way ANOVA)

**Table 4:** Post Hoc Analysis of Ocular Biometric Parameters Among Each Acute Angle-Closure Mechanism Using Tukey’s HSD

Biometric Parameters	CR vs LS [*p* value]^*^	CR vs PB [*p* value]^*^	CR vs PL [*p* value]^*^	LS vs PB [*p* value]^*^	LS vs PL [*p* value]^*^	PB vs PL [*p* value]^*^
ACD (mm)	1.7 vs 1.6 [0.58]	1.7 vs 1.9 [0.45]	1.7 vs 2.2 [< 0.01]	1.6 vs. 1.9 [0.07]	1.6 vs. 2.2 [< 0.001]	1.9 vs 2.2 [0.09]
LT (mm)	5.2 vs 5.0 [0.22]	5.2 vs 5.0 [0.36]	5.2 vs 5.0 [0.83]	5.0 vs 5.0 [0.99]	5.0 vs 5.0 [0.98]	5.0 vs 5.0 [0.99]
VL (mm)	14.9 vs 15.9 [< 0.05]	14.9 vs 15.1 [0.84]	14.9 vs 15.1 [0.93]	15.9 vs 15.1 [0.13]	15.9 vs 15.1 [0.41]	15.1 vs 15.1 [1.00]
AL (mm)	22.3 vs 23.0 [< 0.04]	22.3 vs 22.6 [0.71]	22.3 vs 22.7 [0.75]	23.0 vs 22.6 [0.53]	23.0 vs 22.7 [0.88]	22.6 vs 22.7 [0.99]
LP	4.3 vs 4.1 [0.07]	4.3 vs 4.4 [0.95]	4.3 vs 4.7 [< 0.05]	4.1 vs 4.4 [< 0.05]	4.1 vs 4.7 [< 0.001]	4.4 vs 4.7 [< 0.05]
RLP	1.9 vs 1.8 (< 0.01)	1.9 vs 1.9 [0.99]	1.9 vs 2.1 [0.15]	1.8 vs 2.2 [< 0.05]	1.8 vs 2.1 [< 0.001]	1.9 vs 2.2 [0.16]
LAF	2.32 vs 2.17 [< 0.05]	2.3 vs 2.2 [0.25]	2.3 vs 2.2 [0.62]	2.2 vs 2.2 [0.88]	2.2 vs 2.2 [0.93]	2.2 vs 2.2 [1.00]

*HSD* honest significant difference, *ACD* anterior chamber depth, *LT* lens thickness, *VL* vitreal length, *AL* axial length, *LP* lens position, *RLP* relative lens position, *LAF* lens axial length factor, *CR* crowded-angle, *LS* lens subluxation, *PB* pupillary block, *PL* plateau iris syndrome

^*^ *p*-value represents analysis of all four groups with pairwise comparison

The LP was greater in the PL subgroups compared to the PB, CR, and LS subgroups. The LP was smaller in the LS subgroup compared to the CR, PB, and PL subgroups. No significant difference was found in LP between CR and PB. Overall, there was a trend toward a greater lens position, and from smallest to greatest, the subgroups ranked: LS, CR, PB, and PL. Results regarding RLP were similar to LP (Table 4).

The LAF was greater in the CR subgroup compared to the LS subgroup. The VL was the greatest in the LS subgroup compared to all other subgroups. The AL and LT were not significantly different among the three groups.

The Area under the Receiver Operating Characteristic using an ACD of 1.90 mm to distinguish the PL group from all other groups was 0.846.

Subsequent age- and gender-adjusted analysis of ocular biometric parameters for each acute angle-closure mechanism is presented in Table 5.

**Table 5:** Multinomial logistic regression analysis of ocular biometric parameters among each acute angle-closure mechanism (adjusted for age and gender)

Biometric Parameters	CR vs LS [*p* value]	CR vs PB [*p* value]	CR vs PL [*p* value]	LS vs PB [*p* value]	LS vs PL [*p* value]	PB vs PL [*p* value]
ACD (mm)	1.7 vs 1.6 [0.28]	1.7 vs 1.9 [0.18]	1.7 vs 2.2 [< 0.05]	1.6 vs. 1.9 [< 0.05]	1.6 vs. 2.2 [< 0.05]	1.9 vs 2.2 [0.07]
AL (mm)	22.3 vs 23.0 [< 0.05]	22.3 vs 22.6 [0.27]	22.3 vs 22.7 [0.10]	23.0 vs 22.6 [0.08]	23.0 vs 22.7 [0.66]	22.6 vs 22.7 [0.41]
LP	4.3 vs 4.1 [< 0.05]	4.3 vs 4.4 [0.56]	4.3 vs 4.7 [< 0.05]	4.1 vs 4.4 [< 0.05]	4.1 vs 4.7 [< 0.05]	4.4 vs 4.7 [< 0.05]
RLP	1.9 vs 1.9 [< 0.05]	1.9 vs 1.9 [0.99]	1.9 vs 2.1 [0.06]	1.8 vs 2.2 [< 0.05]	1.8 vs 2.1 [< 0.001]	1.9 vs 2.2 [0.07]
LAF	2.32 vs 2.17 [< 0.05]	2.3 vs 2.2 [0.13]	2.3 vs 2.2 [0.19]	2.2 vs 2.2 [0.16]	2.2 vs 2.2 [0.45]	2.2 vs 2.2 [0.82]

*ACD* anterior chamber depth, *LT* lens thickness, *VL* vitreal length, *AL* axial length, *LP* lens position, *RLP* relative lens position, *LAF* lens axial length factor, *CR* crowded-angle, *LS* lens subluxation, *PB* pupillary block, *PL* plateau iris syndrome

Given the significant differences in ACD, LP, and RLP, we compared these parameters in the four subgroups to their respective fellow eyes. The ACDs of the CR and LS subgroups were significantly shallower compared to the ACDs in their respective fellow eyes (1.7 and 1.6 vs 2.1, *p* < 0.01 for both). The LP and RLP of the CR, LS, and PB subgroups were significantly less than in the fellow eyes [(LP: 4.3 vs 4.6, 4.1 vs 4.6, and 4.4 vs 4.6; *p* = < 0.01 for CR, LS, and PB) and (RLP: 1.9 vs 2.0, 1.8 vs 2.0, and 1.9 vs 2.0; *p* < 0.01 for CR, LS, and PB), respectively].

## Discussion

Prior anterior segment imaging studies have identified several biometric parameters in eyes with AAC that differ from normal or fellow eyes, including a shallow anterior chamber [2, 4, 16], a thicker lens [2, 4, 16, 17], a more anterior lens position [2, 4, 17], and a shorter axial length [2, 4, 16, 17]. However, differences among the individual mechanisms of AAC have not been widely evaluated.

This study showed that AAC eyes have a shallower ACD and a more anterior LP and RLP compared to fellow eyes of AAC and PACS eyes. However, in contrast to previous studies [8, 12], our study did not find significant differences in LT or LAF between AAC eyes and either fellow eyes of AAC or PACS eyes [8, 12], potentially due to our strict exclusion of eyes in which the fellow eye contained an intumescent lens.

ACD may provide a useful clinical tool in the identification of CR and LS. First, CR and LS were the two most prevalent AAC mechanisms, suggesting that, in our study population, an AAC eye is more likely to have CR and LS versus PB and PL. Second, CR and LS demonstrated a significantly shallower ACD compared to the fellow eyes, whereas PL and PB did not. Similarly, albeit not statistically significant, CR and LS exhibited the shallowest ACD among the four AAC subgroups. Third, CR had the smallest AL and the greatest LAF among four groups, suggesting the potential important of small ocular dimensions in identifying CR.

Although PL had the deepest ACD among our subgroups, it was still shallow when compared to the ACD in PL measured in prior studies (2.80–3.14 mm) [18–20]. This surprising finding may be explained firstly by the frequent mixed mechanisms that often underlie AAC. For example, in patients with AAC, increased IOP or recurrent AAC in the presence of a patent peripheral iridotomy may suggest the formation of peripheral anterior synechiae or the influence of non-pupillary block mechanisms [21]. Second, the average age of our subjects (60.2 ± 6.9 years) was greater than in previous reports [18, 22]. As the lens thickens with age, the anterior chamber may become more shallow, thereby leading to a more complex clinical presentation [17, 19]. Overall, our findings corroborate prior studies in demonstrating a greater degree of ACD variability in PL compared to other mechanisms [19, 22–25].

This study had several limitations. First, the results of our study were limited by the characteristics of Ramathibodi Hospital as a referral center. The profiles of the patients likely represent one end of the spectrum of patients with AAC. Second, we analyzed biometric parameters regardless of iridotomy status, though nearly one-half of the patients had undergone iridotomy prior to UBM examination. As such, it was difficult to define all mechanisms responsible for certain AAC eyes. Third, our criteria for exclusion of secondary lens subluxation centered on a history of ocular trauma, as zonular status (e.g. disruption or laxity) could not be determined by slit lamp biomicroscopy or UBM. Fourth, the retrospective nature of this study resulted in exclusion of incomplete patient charts. Fifth, the small sample sizes of the subgroups may not have provided enough statistical power to demonstrate significant differences among mechanisms in terms of AL, LT, and LAF.

## Conclusions

The anterior position of the lens was the important factor for AAC development in our patients. CR was the most contributory mechanism to AAC development among the predisposed eyes. This finding provides a better understanding regarding to the role of the lens in the pathogenesis of acute angle-closure and emphasizes the implications of cataract extraction. In our study, the mean age for subjects with plateau iris was greater than in previous studies, which may have led to a more complex clinical presentation and smaller ACD. Further studies with larger sample sizes are warranted in order to elucidate the overall mechanisms responsible for AAC development in individual patients.

## Abbreviations

AAC: Acute angle closure
ACD: Anterior chamber depth
AL: Axial length
CR: Crowded angle
IOP: Intraocular pressure
LAF: Lens axial length factor
LP: Lens position
LS: Lens subluxation
LT: Lens thickness
PACS: Primary angle closure suspect
PB: Pupillary block
PL: Plateau iris syndrome
RLP: Relative lens position
UBM: Ultrasound biomicroscopy
VL: Vitreous length
